# Cardiac mitochondrial plasticity and thermal sensitivity in a fish inhabiting an artificially heated ecosystem

**DOI:** 10.1038/s41598-019-54165-3

**Published:** 2019-11-28

**Authors:** Nicolas Pichaud, Andreas Ekström, Sophie Breton, Fredrik Sundström, Piotr Rowinski, Pierre U. Blier, Erik Sandblom

**Affiliations:** 1 Department of Chemistry and Biochemistry, Université de Moncton, Moncton, NB E1 A 3E9 Canada; 2 Department of Biological and Environmental Sciences, University of Gothenburg, Gothenburg, 405 30 Sweden; 3 Department of Biology, Université du Québec à Rimouski, Rimouski, QC Canada G5L 3A1; 4 Department of Biological Sciences, Université de Montréal, Montréal, QC H2V 2S9 Canada; 5 Department of Ecology and Genetics, Uppsala University, Uppsala, 752 36 Sweden

**Keywords:** Energy metabolism, Animal physiology

## Abstract

Some evidence suggests that cardiac mitochondrial functions might be involved in the resilience of ectotherms such as fish to environmental warming. Here, we investigated the effects of acute and chronic changes in thermal regimes on cardiac mitochondrial plasticity and thermal sensitivity in perch (*Perca fluviatilis*) from an artificially heated ecosystem; the “Biotest enclosure” (~25 °C), and from an adjacent area in the Baltic Sea with normal temperatures (reference, ~16 °C). We evaluated cardiac mitochondrial respiration at assay temperatures of 16 and 25 °C, as well as activities of lactate dehydrogenase (LDH) and citrate synthase (CS) in Biotest and reference perch following 8 months laboratory-acclimation to either 16 or 25 °C. While both populations exhibited higher acute mitochondrial thermal sensitivity when acclimated to their natural habitat temperatures, this sensitivity was lost when Biotest and reference fish were acclimated to 16 and 25 °C, respectively. Moreover, reference fish displayed patterns of metabolic thermal compensation when acclimated to 25 °C, whereas no changes were observed in Biotest perch acclimated to 16 °C, suggesting that cardiac mitochondrial metabolism of Biotest fish expresses local adaptation. This study highlights the adaptive responses of cardiac mitochondria to environmental warming, which can impact on fish survival and distribution in a warming climate.

## Introduction

The thermal environment dictates the body temperature of ectotherms such as fish, and highly influences all metabolic processes. During long-term habitat temperature changes, functional modifications can occur at different organization levels, allowing organisms to maintain important physiological and metabolic processes that promote survival and performance^1–3^. As most fish are highly dependent on oxygen for different physiological processes, the impact of temperature on the underlying mechanistic aspects of the aerobic metabolism has been suggested to have a role in setting the heat tolerance limits of these animals, thus influencing geographic distribution and population structure^4–6^. Specifically, the capacity of fish to maintain functional homeostasis in highly aerobic tissues such as heart and brain, as the environment warms, has been highlighted as crucial in this regard^5,7,8^. Indeed, the ability of cardiac mitochondria to properly adjust function in response to both acute temperature changes (*i.e*. mitochondrial thermal sensitivity) and chronic thermal adjustments (*i.e*. mitochondrial reversible phenotypic plasticity or irreversible genetic adaptation) could represent an important mechanistic basis dictating aerobic metabolism^7–12^. This idea is further supported by observations that cardiac mitochondrial respiration rates in some fish decrease before, or close to, their upper critical thermal maximum (CT_max_)^7–12^.

Mitochondria are organelles powering the aerobic metabolism by transducing dietary substrates into ATP, using more than 90% of the oxygen consumed by the organism^13^. To achieve this essential process, the electron transport system (ETS) constituted by different enzymatic complexes embedded in the inner mitochondrial membrane transfers electrons to oxygen, thereby generating a proton gradient used by the ATP synthase to phosphorylate ADP to ATP (oxidative phosphorylation, OXPHOS). The ATP produced by OXPHOS provides the vast majority of the energy required for maintaining cardiac muscle contraction, which drives cardiovascular oxygen transport to systemic tissues^14^.

Temperature strongly affects the reactions that drive the production of ATP by the mitochondrial machinery, thus affecting the energy output necessary for animal life itself. Short-term acute warming typically results in increased mitochondrial oxygen consumption rates due to higher activity of the ETS enzymes^15^, which should theoretically augment ATP production to sustain the increasing energy demand of cellular processes. However, mitochondrial membranes are rapidly affected by warming, which increases membrane fluidity and results in increased proton leak that dissipates the proton motive force^1,8,9,16–18^. Thus, at higher temperatures, an increased fraction of oxygen is used to compensate for proton leak instead of driving ATP synthesis^8–10,16^. Adjustments in mitochondrial functions are therefore required for mounting a proper energy production, matching ATP demand and supply, to ensure functional homeostasis following more chronic changes in habitat temperature regimes. Such adjustments can occur as a result of both reversible phenotypic plasticity within individuals (*i.e*. acclimation), as well as local genetic adaptation across generations, and may involve changes in the catalytic capacities, reaction rates or relative content of the ETS enzymes, altering mitochondrial oxidative capacities, as well as ATP turn-over^19^. In addition, the cellular distribution and density of mitochondria are also known to be modified by long-term temperature changes which could alter mitochondrial functions^15,17,20–22^. As such, during prolonged warming (several weeks or more), compensation of mitochondrial functions and/or mitochondrial density can occur, although the level of this compensation can vary among tissues^1,7,23,24^. It is generally accepted that thermal compensation occurs in fish during acclimation, resulting in decreased mitochondrial metabolism at warmer temperatures due to decreased mitochondrial respiration rates, decreased mitochondrial density and remodeling of mitochondrial membranes^8,25^. In contrast, decreasing temperature reduces the production of ATP by mitochondria, and so increased mitochondrial oxidative capacities and mitochondrial density are commonly observed as a compensatory mechanism with cold acclimation or adaptation in ectotherms^8,25–30^.

Here, we assessed the thermal sensitivities of cardiac mitochondrial functions in two thermally distinct populations of European perch (*Perca fluviatilis*). Perch were collected either from the Baltic sea (reference population, 16 °C) or from the “Biotest enclosure” (Biotest population, 25 °C); the latter being a ~1 km^2^ man-made enclosure receiving heated cooling water effluents from the Forsmark nuclear power plant, thus representing a chronically heated aquatic ecosystem. The enclosure has been heated for more than 35 years, keeping the water temperature ~5–10 °C above the surrounding archipelago while maintaining natural diurnal and yearly temperature fluctuations^31^. Previous studies have shown cardiorespiratory and metabolic differences between the two populations including thermal compensation of standard metabolic rate, as well as routine heart rate and cardiac output^11,31–33^. However, whether these differences are due to local adaptation or phenotypic plasticity, and whether altered cardiac mitochondrial functions are associated with these differences is still unknown. We hypothesized that the two perch populations have adapted to their respective thermal environments, resulting in divergences in cardiac mitochondrial functions including thermal sensitivities and acclimation capacity. To test this, groups of Biotest and reference perch were chronically acclimated for 8 months to fixed laboratory conditions roughly representing the summer habitat temperatures of each population (*i.e*. 16 and 25 °C for reference and Biotest perch, respectively), as well as to opposite temperatures (*i.e*. 25 and 16 °C for reference and Biotest perch, respectively). We then evaluated mitochondrial oxygen consumption rates in permeabilized cardiac tissues from both populations assayed at 16 and 25 °C to assess inter-population differences in thermal sensitivities of mitochondrial functions, and whether such differences are due to local adaptation or reversible acclimation. Moreover, enzymatic activities of lactate dehydrogenase (LDH) and citrate synthase (CS), markers of aerobic and anaerobic capacities, respectively, were also evaluated. We specifically predicted that if population differences in mitochondrial functions were due to phenotypic plasticity, this would translate in thermal compensation with both populations displaying similar respiration rates at the same acclimation temperatures and responding similarly to acute assay temperature changes. However, if populations exhibited local thermal adaptation, this would result in population divergences in mitochondrial functions and acute thermal sensitivity at one or both acclimation temperatures.

## Results

### Morphological variables

Following the 8-month laboratory acclimation, body mass (M_b_), fork length (FL), condition factor (CF), as well as relative ventricular mass (RVM) were all influenced by the acclimation temperature (Table 1). M_b_ and CF were higher in both populations when fish were acclimated to 25 °C (P-values < 0.025 for M_b_; P-values < 0.006 for CF; Table 1), whereas the FL was only significantly higher in reference fish acclimated to 25 °C (P = 0.002; Table 1). Moreover, the RVM was higher in fish from both populations acclimated to 16 °C compared to fish acclimated to 25 °C (P-values < 0.025; Table 1). In contrast to the situation when fish were collected in the wild (see Materials and Methods), no significant differences were detected for M_b_, FL, CF and RVM between reference and Biotest fish after 8-month acclimation to the same temperature.

**Table 1:** Morphological variables of perch (*Perca fluviatilis*) after long-term thermal acclimation.

Population	Acclimation Temperature	M_b_ (g)	FL (mm)	CF	RVM (%)
**Reference**	16 °C (n = 10)	21.06 ± 2.76^a^	122.1 ± 5.0^a^	1.10 ± 0.02^a^	0.0679 ± 0.0011^a^
	25 °C (n = 10)	35.09 ± 2.24^b^	142.2 ± 3.3^b^	1.21 ± 0.03^b^	0.0630 ± 0.0009^b^
**Biotest**	16 °C (n = 11)	25.51 ± 2.02^a^	131.2 ± 2.8^a,b^	1.11 ± 0.02^a^	0.0676 ± 0.0014^a^
	25 °C (n = 12)	37.39 ± 2.02^b^	144.2 ± 3.0^b^	1.23 ± 0.01^b^	0.0630 ± 0.0008^b^

Dissimilar letters denote significant differences between the different acclimated groups. M_b_: body mass; FL: fork length; CF: condition factor; RVM: relative ventricular mass.

### Cardiac mitochondrial respiration rates and mitochondrial ratios

Mitochondrial oxygen consumption rates were assayed at two different assay temperatures, 16 and 25 °C, in permeabilized cardiac tissue of perch from both populations that were acclimated for 8 months in laboratory conditions at either 16 or 25 °C. For each mitochondrial parameter measured, the effects of population, acclimation temperature, assay temperature as well as the interactions between these different factors were evaluated (Table 2).

**Table 2:** Analyses of variance showing F ratios for perch (*Perca fluviatilis*) after long-term thermal acclimation.

	*DF*	Population *df* = 1	Assay Temperature *df* = 1	Acclimation Temperature *df* = 1	Population × Assay Temperature *df* = 1	Assay Temperature × Acclimation Temperature *df = *1	Population × Acclimation Temperature *df* = 1	Population × Assay Temperature × Acclimation Temperature df = 1	Co-variate Weight *df* = 1
**Mitochondrial respiration rates**									
CI-LEAK	34	3.58	32.73***	2.84	8.93***	15.11***	10.44**	21.39***	5.84*
CI-OXPHOS	34	0.75	3.53	10.28**	0.23	6.26*	4.12	4.73*	0.84
CI + CII-OXPHOS	34	0.45	2.36	11.59**	0.27	3.81	4.34*	3.47	0.50
CI + CII-ETS	34	0.64	0.97	14.37***	0.18	0.93	7.26*	1.55	0.01
Complex IV	34	0.51	3.10	13.93***	1.07	2.57	4.69*	7.97**	0.21
P_I_/L_I_ ratio	34	12.48**	24.42***	23.64***	9.82**	7.22*	31.47***	12.27**	2.60
E_I+II_/P_I+II_ ratio	34	0.18	8.18**	1.04	0.32	25.22***	5.59*	10.82**	8.42**

*P < 0.05; **P < 0.01; ***P < 0.001.

*DF*: denominator degree of freedom*; df*: numerator degree of freedom.

#### Differences between populations acclimated to 16 °C

**Figure 1:**
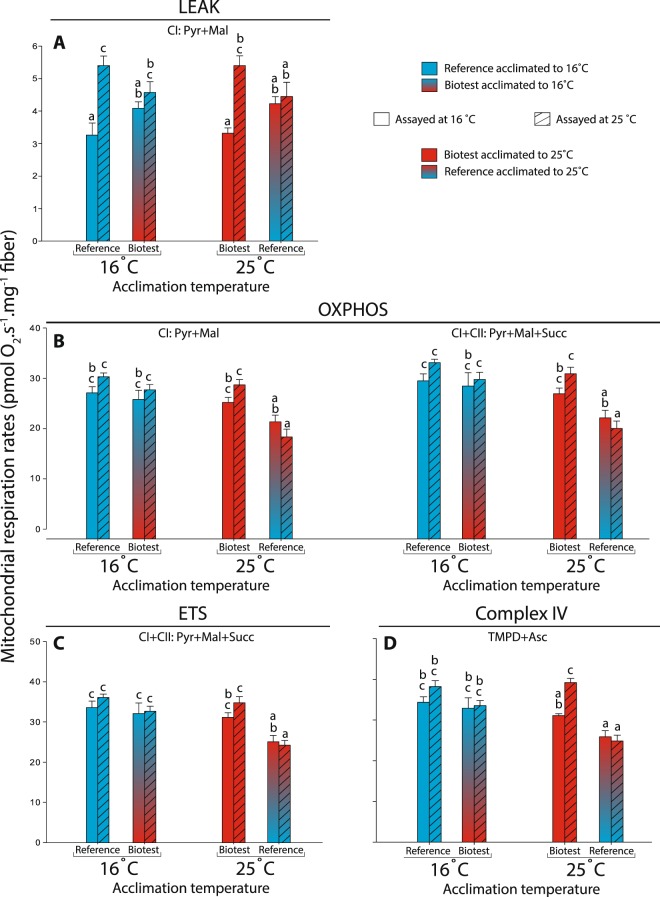
Mitochondrial respiration rates in permeabilized cardiac fibers of perch (*Perca fluviatilis*) after long-term thermal acclimation. Mitochondrial respiration rates were measured during A) the LEAK respiration in presence of pyruvate + malate (CI-LEAK); B) the OXPHOS respiration after addition of ADP (CI-OXPHOS) and succinate (CI + CII-OXPHOS); C) the uncoupled (ETS) respiration after injection of FCCP (CI + CII-ETS); and D) with TMPD + ascorbate + cytochrome c (Complex IV) after inhibition of complexes I and III. N = 5–6 for each population at each assay temperature. Results are means ± s.e.m. Dissimilar letters represent significant differences among groups (P < 0.05).

**Figure 2:**
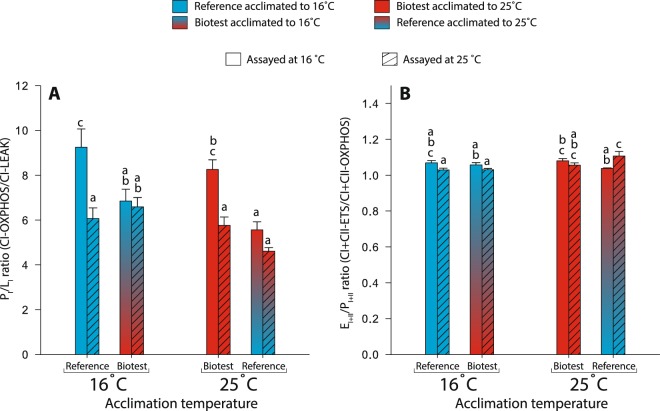
Mitochondrial ratios calculated from respiration rates measured in permeabilized cardiac fibers of perch (*Perca fluviatilis*) after long-term thermal acclimation. (**A**) P_I_/L_I_ ratio was taken as an indicator of mitochondrial integrity and mitochondrial coupling and was calculated as CI-OXPHOS/CI-LEAK; (**B**) E_I + II_/P_I + II_ was determined to estimate the ETS reserve capacity and was calculated as CI + CII-ETS/CI + CII-OXPHOS. N = 5–6 for each population at each assay temperature. Results are means ± s.e.m. Dissimilar letters represent significant differences among groups (P < 0.05).

Cardiac tissue from fish of both populations acclimated to 16°C displayed very similar respiration rates (CI-LEAK, CI-OXPHOS, CI + CII-OXPHOS, CI + CII-ETS and Complex IV) when assayed at both 16 and 25 °C (Fig. 1). The P_I_/L_I_ ratio at the level of complex I (CI-OXPHOS/CI-LEAK), which is an indicator of mitochondrial quality and of mitochondrial coupling^10,34^, was significantly decreased in Biotest fish compared to reference fish when assayed at 16 °C (P = 0.024; Fig. 2A), but was similar when assayed at 25 °C. Moreover, the E_I+II_/P_I+II_ ratio, which represents the ETS reserve capacity and indicates a possible limitation of the phosphorylation system if higher than 1.0^10,34^, was similar between populations at both assay temperatures (Fig. 2B).

#### Differences between populations acclimated to 25 °C

When acclimated to 25 °C, there were no differences in CI-LEAK between populations at either of the assay temperatures (Fig. 1A). However, the reference fish tissues displayed consistently lower CI-OXPHOS, CI + CII-OXPHOX, CI + CII-ETS and Complex IV respiration rates compared to Biotest tissues when assayed at 25 °C (all P-values < 0.001; Fig. 1B–D). Moreover, Biotest fish displayed higher P_I_/L_I_ ratios than reference fish when measured at 16 °C (P = 0.003, Fig. 2A), while the E_I+II_/P_I+II_ ratios were similar between populations at either of the assay temperatures (Fig. 2B).

#### Effects of the different thermal acclimation regimes within populations

The cardiac tissues from Biotest fish displayed almost identical respiration rates between acclimation temperatures across both assay temperatures (Fig. 1; Table 2). Moreover, the mitochondrial ratios (both P_I_/L_I_ and E_I+II_/P_I+II_) were also similar between acclimation temperatures when assayed at either 16 °C or 25 °C in Biotest tissues (Fig. 2; Table 2).

CI-LEAK assayed at 25 °C was higher in tissues from reference fish acclimated to 16 °C than in those acclimated to 25 °C (P = 0.041; Fig. 1A; Table 2). Moreover, cardiac tissues from the reference population acclimated to 25 °C displayed consistently lower respiration rates compared to those acclimated to 16 °C. In this case, significant differences were detected for CI + CII-OXPHOS, CI + CII-ETS and Complex IV when assayed at 16 °C (P = 0.033, P = 0.012, and P = 0.014, respectively; Fig. 1B–D) and for CI-OXPHOS, CI + CII-OXPHOS, CI + CII-ETS and Complex IV when assayed at 25 °C (P < 0.001, P < 0.001, P = 0.002, and P < 0.001, respectively; Fig. 1B–D). Tissues from reference fish acclimated to 16 °C also displayed higher P_I_/L_I_ ratios compared to reference fish acclimated to 25 °C when assayed at 16 °C (P < 0.001; Fig. 2A). Moreover, reference fish acclimated to 25 °C had higher E_I+II_/P_I+II_ values assayed at 25 °C than reference fish acclimated to 16 °C (P < 0.001; Fig. 2B).

#### Differences between populations acclimated to their respective natural habitat temperatures

When both populations were acclimated to temperatures close to their natural environment (*i.e*. reference acclimated to 16 °C and Biotest acclimated to 25 °C), there were no differences detected for all mitochondrial respiration rates assayed at either 16 °C or 25 °C (Fig. 1; Table 2). Similarly, no differences were detected in the mitochondrial ratios (both P_I_/L_I_ and E_I+II_/P_I+II_) when assayed at 16 °C or when assayed at 25 °C (Fig. 2; Table 2).

#### Differences between reference fish acclimated to 25 °C and Biotest fish acclimated to 16 °C

No differences were detected for CI-LEAK when comparing cardiac tissues from reference perch acclimated to 25 °C with Biotest fish acclimated to 16 °C, when assayed at either 16 or 25 °C (Fig. 1A; Table 2). However, reference fish tissues displayed significantly lower CI + CII-ETS and Complex IV when assayed at 16 °C (P = 0.049 and P = 0.046, respectively; Fig. 1B–D), as well as lower CI-OXPHOS, CI + CII-OXPHOS, CI + CII-ETS and Complex IV when assayed at 25 °C compared to Biotest fish tissues (P < 0.001, P = 0.002, P = 0.026 and P = 0.021, respectively; Fig. 1B–D).

For the P_I_/L_I_ ratio, cardiac tissues from reference fish acclimated to 25 °C and Biotest fish acclimated to 16 °C displayed similar values at either of the assay temperatures (Fig. 2A). However, for the E_I+II_/P_I+II_ ratio, a significant increase was detected in tissues from reference fish acclimated to 25 °C compared to Biotest fish acclimated to 16 °C, but only when assayed at 25 °C (P < 0.001; Fig. 2B).

#### Effects of assay temperatures within populations

In cardiac tissues from both reference and Biotest populations acclimated to temperatures reflecting their natural habitat temperatures (*i.e*. 16 and 25 °C, respectively), CI-LEAK was significantly higher when assayed at 25 °C (both P-values˂0.001; Fig. 1A; Table 2). However, when reference and Biotest perch were acclimated to 25 and 16 °C, respectively, the assay temperatures had no effects on CI-LEAK (Fig. 1A, Table 2). Surprisingly, the CI-OXPHOS and CI + CII-OXPHOS respiration rates of tissues from both populations acclimated to either temperature regimes did not differ significantly between assay temperatures (Fig. 1B; Table 2). Similarly, the CI + CII-ETS was not different between assay temperatures for tissues from either of the different acclimation groups (Fig. 1C; Table 2).

As for the mitochondrial ratios, a significant reduction of P_I_/L_I_ was observed with increased assay temperature in tissues from both reference fish acclimated to 16 °C and Biotest fish acclimated to 25 °C (P˂0.001 and P = 0.008, respectively). However, no such effects were observed in tissues from reference and Biotest fish acclimated to 25 and 16 °C, respectively (Fig. 2A; Table 2). For E_I+II_/P_I+II_, all the different groups had a relatively small ETS reserve capacity (between 1.03 and 1.11). The only significant difference detected for this ratio between the assay temperatures was in reference fish acclimated to 25 °C that exhibited higher reserve capacity when measured at 25 °C compared to 16 °C (P = 0.002; Fig. 2B; Table 2).

Finally, for Complex IV, the only difference between assay temperatures was observed in tissues from Biotest fish acclimated to 25 °C which displayed higher complex IV respiration rates when measured at 25 °C (P = 0.011; Fig. 1D; Table 2).

To further elucidate the effects of assay temperatures, we calculated the temperature coefficients (Q_10_) for each mitochondrial parameter assayed between 16 and 25 °C. The highest Q_10_ observed with acute temperature change was for CI-LEAK in tissues from the populations following acclimation to their habitat temperatures. Q_10_ values for reference fish acclimated to 16 °C and for Biotest fish acclimated to 25 °C were 1.75 and 1.72, respectively, whereas for reference fish acclimated to 25 °C and for Biotest fish acclimated to 16 °C, Q_10_ values were much lower (1.06 and 1.14, respectively; Table 3). For the other mitochondrial respiration rates, the Q_10_ measured between assay temperatures was much lower in both populations acclimated to the temperatures reflecting their respective habitat temperatures (1.08–1.13 for reference fish acclimated to 16 °C, and 1.13–1.29 for Biotest fish acclimated to 25 °C; Table 3), and even below 1.0 when the two populations were acclimated to the opposite environmental temperature regimes (Table 3).

**Table 3:** Q_10_ values calculated from mean cardiac mitochondrial respiration rates measured at 16 and 25 °C in perch (*Perca fluviatilis*) acclimated to different thermal regimes.

Population	Acclimation Temperature	CI-LEAK	CI-OXPHOS	CI + CII-OXPHOS	CI + CII-ETS	Complex IV
Reference	16 °C (n = 10)	1.75	1.12	1.13	1.08	1.13
	25 °C (n = 10)	1.06	0.86	0.90	0.96	0.95
Biotest	16 °C (n = 11)	1.14	1.08	1.05	1.02	1.02
	25 °C (n = 12)	1.72	1.14	1.16	1.13	1.29

### Enzymatic activities of CS and LDH

**Figure 3:**
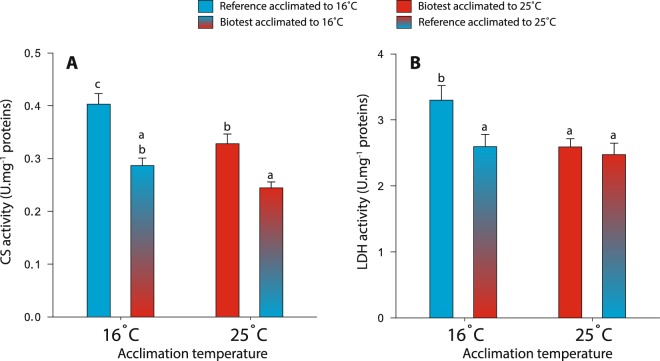
Enzymatic activities of citrate synthase and lactate dehydrogenase in cardiac tissues of perch (*Perca fluviatilis*) after long-term thermal acclimation. Results are presented as means ± s.e.m and illustrate A) citrate synthase (CS) and B) lactate dehydrogenase (LDH) activities measured at room temperature and expressed as U.mg^−1^ proteins, where U is 1 µmol of substrate transformed per minute. N = 10–12 for each acclimation group. Dissimilar letters represent significant differences among groups (P < 0.05).

CS and LDH were measured at room temperature to evaluate changes in the enzymatic content associated with aerobic and anaerobic capacities, respectively, in the different acclimation groups. Both enzymes were influenced by the acclimation temperature (F_1,39_ = 9.94, P = 0.003 for CS; F_1,39_ = 4.90, P = 0.033 for LDH) and by the interaction between population and acclimation temperature (F_1,39_ = 37.69, P < 0.001 for CS; F_1,39_ = 5.54, P = 0.024 for LDH). When acclimated to 16 °C, reference fish displayed significantly higher cardiac CS and LDH activities than Biotest fish at the same acclimation temperature, as well as Biotest and reference fish acclimated to 25 °C (for CS: P < 0.001, P = 0.014, and P < 0.001, respectively; for LDH: P = 0.042, P = 0.036, and P = 0.013, respectively; Fig. 3A,B). Moreover, Biotest fish acclimated to 25 °C had higher CS activity than reference fish acclimated to the same temperature (P = 0.003; Fig. 3A).

## Discussion

The present study was designed to evaluate if, and to what extent, 35 years of chronic habitat warming in a perch population has been sufficient to allow fixation of key heart mitochondrial traits that could be associated with adaptation to a warmer thermal habitat. Using fixed temperature conditions, we were able to investigate population differences due to adaptation to their natural habitat temperatures and if cardiac mitochondrial functions could have participated in these adaptations.

### Acute thermal sensitivity of mitochondrial functions

Considering the two assay temperatures tested, our results suggest that both populations exhibit higher acute mitochondrial thermal sensitivity, as indicated by the higher Q_10_ values across acute assay temperatures, when they were acclimated to temperatures mimicking those from their natural habitat. The CI-LEAK respiration rates of reference and Biotest fish acclimated to their respective habitat temperatures displayed the highest thermal sensitivity (Q_10_ = 1.75 and 1.72, respectively), with significantly higher CI-LEAK when measured at 25 °C compared to those measured at 16 °C. As a result, the P_I_/L_I_ ratios were diminished when measured at 25 °C for these acclimation groups. This was expected and likely reflect that a greater fraction of oxygen is consumed by mitochondria at higher assay temperatures due to increased proton leak through the inner mitochondrial membrane^1,8–10,16^. Alternatively, it has been shown that an upsurge of reactive oxygen species (ROS) occurs in heart mitochondria of fish exposed to acute warming^12^_._ As increased proton leak can help to attenuate ROS production^35^, the higher thermal sensitivity observed for CI-LEAK could be a response to mitigate an upsurge of ROS during acutely elevated environmental temperatures. When reference fish were acclimated to 25 °C or when Biotest fish were acclimated to 16 °C, the Q_10_’s dropped to 1.06 and 1.14, respectively, suggesting a loss of mitochondrial thermal sensitivity. This was also detected with the other respiration rates measured, and most strikingly in reference fish acclimated to 25 °C that displayed the lowest acute Q_10_ (0.86 for CI-OXPHOS).

### Limitations of mitochondrial respiration due to acute temperature changes

Interestingly, reference fish acclimated to 25 °C also displayed the highest E_I+II_/P_I+II_ ratio measured at 25 °C indicating a greater ETS reserve capacity, which is in accordance with another study on cardiac tissues of killifish, *Fundulus heteroclitus*, showing that this parameter is affected by assay temperature^36^. At the level of complex I, this also coincided with the lowest P_I_/L_I_ ratio measured at 25 °C. These results in reference fish acclimated to 25 °C suggest that mitochondrial substrate oxidation capacity at the level of complex I is limiting mitochondrial respiration, resulting in increased ETS reserve capacity. Indeed, Chung and Schulte (2015) observed in liver tissues from *F. heteroclitus* that acclimation to 33 °C induced a suppression of mitochondrial respiration rates associated with substrates supplying electrons to complex I^23^. However, this contrasts with other studies on the heart of killifish which found that the decreased mitochondrial respiration rates following high-temperature acclimation were not specific to complex I^7,36^. An alternative explanation is that the mitochondrial thermal tolerance of reference fish acclimated to 25 °C is not constrained by mitochondrial oxidative capacities of complex I, but rather by oxidative capacities of enzymes upstream of complex I. While previous work on the enzymatic activities measured in individuals of both perch populations sampled in the field showed that tricarboxylic acid cycle enzymes such as pyruvate dehydrogenase and citrate synthase have the same thermal sensitivities when assayed between 23 and 30 °C^15^, no information is available about these enzymes when the fish were acclimated to different temperatures.

A reduced acute thermal sensitivity has also been reported in heart and skeletal muscle of several fish species, at extreme temperatures close to their upper thermal tolerance ranges (*i.e*. CT_max_)^12,16,37^. The CT_max_ of reference perch at the whole animal level has been shown to be around 29.8 °C when acclimated to 18 °C^38^, and should theoretically be slightly higher when the fish are acclimated to 25 °C^39,40^. The current observation suggests that the cardiac mitochondrial metabolism may have reached its maximum capacity in reference fish acclimated to 25 °C, as thermal independence (acute Q_10_ near 1.0) or negative thermal dependence (acute Q_10_ below 1.0) were observed. It is thus likely that higher assay temperatures could result in dramatically decreased mitochondrial respiration rates and thus cardiac ATP production before CT_max_ is reached as previously suggested^9^. However, measurements of cardiac mitochondrial functions at higher assay temperatures in these fish are necessary to verify this assumption.

### Acclimation capacity of mitochondrial functions in reference fish

Reference perch acclimated to 25 °C clearly displayed decreased mitochondrial functions at both assay temperatures compared to the other groups. These steady reductions of most mitochondrial respiration rates (except CI-LEAK) in reference perch acclimated to 25 °C could result from thermal metabolic compensation as it has been shown to occur with increased acclimation temperatures in several tissues (including cardiac tissues) of different species of fish^23–25^. Thus, reference fish might be able to offset the thermodynamic effects of chronically warmer temperatures via phenotypic plasticity, by reducing their mitochondrial capacities while still maintaining the same proton motive force and proper ATP production. However, measurements of mitochondrial membrane potential as well as ATP production are required to confirm this hypothesis. Thermal acclimation could also result in adjustment of mitochondrial density^25,30,41,42^, for which the activity of CS is typically used as a proxy^10,43^. CS as well as LDH (marker of anaerobic capacity), also displayed patterns of metabolic compensation, with reference fish acclimated to 16 °C having higher CS and LDH activities than those acclimated to 25 °C. Thus, warm acclimation in reference fish seems to have resulted in decreased mitochondrial density, which in turn could explain the reduced mitochondrial rates observed in this group.

### Mitochondrial adaptation to warmer habitat in Biotest fish

Fish from the Biotest population had similar respiration rates, as well as CS and LDH activities, at both acclimation temperatures. Although respiration rates for Biotest perch acclimated to either 16 or 25 °C were also similar to reference perch acclimated to 16 °C, the response of the measured enzymes to acclimation was different, as reference fish acclimated to 16 °C displayed higher CS and LDH activities than the other groups. Moreover, it has been shown that slight but highly significant genetic differentiation has evolved in the Biotest population, albeit only a small number of loci were examined^44^. Altogether, this suggests that Biotest perch display local adaptation allowing for maintaining mitochondrial functions in their warmer habitat, as acclimation to 16 °C for 8 months did not result in patterns of thermal compensation. Alternatively, this adaptive effect could also be the result of developmental programming. Indeed, it has been suggested that environmental conditions during development may influence the offspring phenotype expression, resulting in a better preparation for environmental circumstances that the organism is likely to encounter during its life^2,45–47^. It has been shown that Biotest perch spawn and reach maturity earlier, and generally grow larger and faster at early ages^31,48,49^, suggesting developmental programming to warmer temperatures in this population^47,50^. Considering that the fish used in our study were young-of-the-year perch at the time of capture, it is possible that the effects of acclimation temperatures observed on mitochondrial metabolism were the result of developmental plasticity divergences between the Biotest and the reference populations (likely through epigenetic changes^47,50^). Determining DNA methylation patterns and/or histone acetylation in both populations at different life stages could shed light on such programming effect.

## Conclusions

The current data demonstrate that Biotest and reference populations originating from two thermally distinct habitats display clear differences in their cardiac mitochondrial thermal sensitivity and plasticity. While cardiac tissues from both populations displayed higher mitochondrial acute thermal sensitivities when acclimated to temperatures close to their natural habitats, these mitochondrial sensitivities were reduced in Biotest and reference populations when acclimated to colder (16 °C) and warmer (25 °C) thermal conditions, respectively. In terms of acclimation capacities, our results suggest that the two populations might have different strategies to adjust their mitochondrial metabolism. While reference perch display evidence of thermal compensation (phenotypic plasticity), the Biotest population might have mitochondria adapted to their warmer habitat due to genetic adaptation, developmental plasticity, or both. However, future experiments focusing on the acclimation capacities of both populations to higher temperatures and at different life stages are needed to verify this. In conclusion, the current findings show that in the Biotest population, 35 years of evolution have resulted in effective adjustments of the mitochondrial functions to warmer temperatures, facilitating adaptation to the new thermal habitat. Thus, we suggest that selection for specific mitochondrial phenotypes are likely to occur with climate warming. However, it is likely that in the face of extreme thermal events predicted with climate warming such as longer and more frequent transient heat waves, mitochondrial functions of fish populations will probably not have time to adjust, ultimately resulting in a loss of homeostasis and decreased survival.

## Methods

### Experimental animals and holding conditions

Perch were caught between the 25^th^ of August and the 11^th^ of September in 2013 by hand trawl in four different locations inside the Biotest enclosure (23.1 ± 2.0 °C) and four different locations in the reference area (17.6 ± 0.4 °C) that were at least 3 km away from the location where Biotest water discharges into the Baltic sea area (N = 30 for each population). These fish were mainly young-of-the-year *P. fluviatilis* at the time of capture with a mean body mass of 7.2 ± 0.4 g and 11.9 ± 0.5 g and a mean total length of 72.9 ± 1.7 mm and 95.9 ± 1.3 mm for reference and Biotest fish, respectively. The perch were then transported to the laboratory at Uppsala University (Sweden), where they were held in 250 L tanks supplied with aerated freshwater and kept at a 12:12 h diurnal light:dark cycle. Fish from the reference and Biotest populations (N = 15 per holding tank) were either acclimated to temperatures close to their natural habitat temperatures, or to 25 and 16 °C, respectively, for 8 months. During the acclimation period, fish were fed with frozen chironomids *ad libitum* 1–2 times per day until three days before being used for experiments between the 23^rd^ and the 27^th^ of April 2014. During the acclimation period, the mortality rates for the different groups were as follow: 7% (2 fish) for reference fish acclimated to 16 °C, 17% (5 fish) for reference fish acclimated to 25 °C, 3% (1 fish) for Biotest fish acclimated to 16 °C, and 10% (3 fish) for Biotest fish acclimated to 25 °C. All experiments were performed in agreement with the ethical permits 65–2012 and C176/12 from the animal ethics committees in Gothenburg and Uppsala (Sweden), respectively.

### Tissue sampling and morphological variables

Fish were netted from the holding tanks and killed with a sharp cranial blow. For all fish, body mass (M_b_) and fork length (FL) were determined. The heart was then quickly excised, and the ventricle was dissected free, blotted and the ventricle mass (M_v_) was determined.

The relative ventricular mass (RVM) was calculated as:

$${\rm{RVM}}={{\rm{M}}}_{{\rm{v}}}/{{\rm{M}}}_{{\rm{b}}}.$$

The fish condition factor (CF) was calculated as:

$${\rm{CF}}={(\mathrm{100M}}_{{\rm{b}}})/{{\rm{FL}}}^{{\rm{3}}}$$

with M_b_ and M_v_ in g and FL in cm.

The ventricle was cut in half and each part was either directly placed in ice-cold relaxing solution (2.77 mM CaK_2_EGTA, 7.23 mM K_2_EGTA, 5.77 mM Na_2_ATP, 6.56 mM MgCl_2_, 20 mM Taurine, 15 mM Na_2_phosphocreatine, 20 mM imidazole, 50 mM MES,0.5 mM dithiothreitol, pH 7.1) for mitochondrial respiration experiments, or transferred to liquid nitrogen and kept at −80 °C for further enzymatic assays.

### Cardiac mitochondrial respiration rates and mitochondrial ratios

Permeabilization of cardiac muscle fibers on the ventricle and respirometry were performed to assess mitochondrial respiration as described elsewhere^11,51^. Reference and Biotest perch were acclimated to both 16 and 25 °C and assayed at both 16 and 25 °C, with N = 5–6 for each treatment group at each assay temperature. The permeabilized fibers from the ventricle were blotted and weighed (3.5–8.7 mg) using a Sartorius BP1 10 S with 0.1 mg readability (Sartorius, Göttingen, Germany). Next, fibers were placed into glass mini chambers (Loligo® Systems ApS, Tjele, Denmark) equipped with oxygen sensor spots OXSP5 (Pyro Science GmbH, Aachen, Germany) fixed on the inner surface wall, and the oxygen concentration was measured using FireStingO_2_ probes connected to a FireStingO_2_ fiber-optic oxygen meter (Pyro Science GmbH, Aachen, Germany), as previously described^11,51^. After the chambers were closed, a substrate-uncoupler-inhibitor titration (SUIT) protocol was performed as previously described^11^ using: (i) pyruvate and malate (5 mM and 0.5 mM respectively) to measure the leak (non-phosphorylating) state for complex I (CI-LEAK); (ii) + ADP (5 mM) to monitor the phosphorylating state for complex I (CI-OXPHOS); (iii) + succinate (10 mM) to assess maximum phosphorylating state with convergent electrons from complex I and complex II (CI + CII-OXPHOS); (iv) + FCCP (titration of 0.25 µM steps) to trigger uncoupled respiration and measure the ETS maximum capacity (CI + CII-ETS); (v) + rotenone (1 µM) + antimycin A (2.5 µM) to inhibit complexes I and III, and measure residual oxygen consumption which was used to correct all the mitochondrial respiration rates; and (vi) Ascorbate (2 mM) + TMPD (0.5 mM) were added after raising the oxygen concentration in the chamber to evaluate the maximum capacity of complex IV. Cytochrome c (10 µM) was then added to estimate the outer mitochondrial membrane integrity of the permeabilized tissue^52^. All preparations denoted less than 8% increase in oxygen consumption after addition of cytochrome c, denoting functional integrity of the outer mitochondrial membrane^52^. Finally, sodium azide (100 mM) was injected to completely inhibit complex IV and calculate the auto-oxidation of TMPD to correct Complex IV respiration rates^53^. All measurements are presented as means of mass-specific mitochondrial respiration rates expressed as pmol O_2_.s^−1^.mg^−1^ of permeabilized fibers ± s.e.m.

CI-LEAK and CI-OXPHOS were used to calculate the P_I_/L_I_ ratio at the level of complex I (CI-OXPHOS/CI-LEAK) which is an indicator of mitochondrial quality and of mitochondrial coupling^10,34^. CI + CII-OXPHOS and CI + CII-ETS were used to calculate the E_I+II_/P_I+II_ ratio which represents the ETS reserve capacity and indicates a possible limitation of the phosphorylation system if higher than 1.0^10,34^.

### Temperature coefficients (Q_10_) of mitochondrial respiration rates

Temperature coefficients of mitochondrial respiration were calculated according to the van’t Hoff equation:

Q_10_ = $${({{\rm{R}}}_{2}/{{\rm{R}}}_{1})}^{10/({{\rm{T}}}_{2}-{{\rm{T}}}_{1})}$$

where R_1_ represents mean of the mitochondrial respiration rates measured at assay temperature T_1_ (16 °C), and R_2_ represents mean of the mitochondrial respiration rates measured at assay temperature T_2_ (25 °C).

### Enzymatic activities of CS and LDH

Frozen ventricles were homogenized in 50 mM potassium phosphate buffer, pH 7.2. The homogenates were centrifuged at 500 g for 5 min at 4 °C, and the supernatant was used for the determination of CS and LDH activities, following existing protocols^15,54^. Both enzymes were measured in triplicates using a microplate reader (SpectraMax 290 microplate reader, Molecular Devices, Sunnyvale, CA, USA) at room temperature. While this approach did not allow us to infer the direct effects of assay temperature on enzymatic function, changes in their activities still provide a good general proxy for changes in mitochondrial density and aerobic capacity (CS), and anaerobic (LDH) enzymatic capacities during acclimation^11,43^. Briefly, the activity of CS (EC 4.1.3.7) was measured by following the reduction of 5,5′-dithiobis-2-nitrobenzoic acid at 412 nm in presence of AcetylCoA and oxaloacetic acid, while LDH activity (EC 1.1.1.27) was determined by following the disappearance of NADH at 340 nm in presence of pyruvate. Both enzymatic activities were normalized by protein content following the bicinchoninic acid method^55^ and are expressed as U.mg^−1^ proteins ± s.e.m., where U is 1 µmol of substrate transformed per minute.

### Statistical analysis

All statistical analyses were performed with R software (version 3.1.0, Free Software Foundation, Boston, MA, USA). For all the parameters measured, data were fitted to a linear model with body mass as covariate. Normality of residuals was checked, homogeneity of variances was verified using the Levene’s test, and data were transformed when required. Mitochondrial respiration rates and mitochondrial ratios were analyzed using a three-way ANOVA (type III), in which population (Biotest and Reference), assay temperature (16 and 25 °C), and acclimation temperature (16 and 25 °C) were included as fixed effects, and interactions between these different experimental factors were tested. For enzymatic activities of CS and LDH, a two-way ANOVA (type II) was performed with population (Biotest and Reference) and acclimation temperature (16 and 25 °C) as fixed factors, including the population × acclimation temperature interaction. In all cases, multiple comparisons were tested with pairwise comparisons of the least-squares means using adjusted P-values (Tukey method) with significance set at P < 0.05.

### Ethical approval

Experimental protocols were approved and performed in agreement with the ethical permits 65–2012 and C176/12 from the animal ethics committees in Gothenburg and Uppsala (Sweden), respectively.

## Electronic supplementary material

Supplementary Information

## Data Availability

The datasets for this manuscript has been uploaded as part of the supplementary material.
